# Supplementary material for: Diagnostic accuracy evaluation of a point-of-care antigen test for SARS-CoV-2 and influenza in UK primary care (RAPTOR-C19)
Source: PLoS One. 2025 Aug 7;20(8):e0329611. doi: 10.1371/journal.pone.0329611 (PMC12331028; doi:10.1371/journal.pone.0329611)
Supplement: S1 Table — (DOCX) [file pone.0329611.s001.docx]

**S1 Table.** **Primers and probes for the detection of Influenza A & B viruses by rtRTPCR.**

Probes AH1pdm09 Probe 3 and AH3 Probe (2023/24) (marked with asterisks) replaced probes H1pmd09 1 & 2 and AH3 probe (2021/22) respectively for the November 2023 to March 2024 recruitment period.

^1^Ellis J, Curran M. 2001 Simultaneous molecular detection and confirmation of influenza AH5, with internal control. Methods Mol Biol. 2011;665:161-81

^2^Terrier O, et al 2011. Cellular transcriptional profiling in human lung epithelial cells infected by different subtypes of influenza A viruses reveals an overall down-regulation of the host p53 pathway. Virol J 8:285 All other primers/probes designed in-house

| Target | Target Gene | Oligonucleotide | Sequence | Final Concentration (nM) |
| --- | --- | --- | --- | --- |
| Influenza A | M | AM Forward | GAG TCT TCT AAC MGA GGT CGA AAC GTA ^1^ | 900 |
|  |  | AM Reverse | GGG CAC GGT GAG CGT RAA ^1^ | 900 |
|  |  | AM Probe | JUN - TCA GGC CCC CTC AAA GCC GAG - QSY ^2^ | 250 |
| Influenza A (H1N1)pdm09 | HA | AH1pdm09 Forward | TTA CCA GAT TTT GGC RAT CTA YT | 600 |
|  |  | AH1pdm09 Reverse | CCA GGG AGA CTA SCA RYA CCA | 600 |
|  |  | AH1pdm09 Probe 1 | 6FAM – ACW GTY GCC AGT TC - MGBNFQ | 120  (combined 1:1) |
|  |  | AH1pdm09 Probe 2 | 6FAM – ACW GCY GCC AGT TC - MGBNFQ |  |
|  |  | *AH1pdm09 Probe 3 (2023/24) | 6FAM – ACT GYY GCC AGY TC - MGBNFQ | 225 |
| Influenza A H3N2 | HA | AH3 Forward | TGG GAC CTT TTY GTT GAA MG | 400 |
|  |  | AH3 Reverse | CGG ATG AGG CAA CTA GTG AYC TA | 500 |
|  |  | AH3 Probe (2020/21) | VIC - CCW ACA GCA ACT GTT AYC - MGBNQF | 20 |
|  |  | AH3 Probe (2021/22) | VIC - CCW ACA GCA RCT GTT AYC - MGBNQF | 250 |
|  |  | *AH3 Probe (2023/24) | VIC - CCW ACA GYA RCT GTT AYC - MGBNFQ | 250 |
| Influenza B | NP | BNP Forward | GCA GCT CTG ATG TCC ATC AAG CT ^1^ | 400 |
|  |  | BNP Reverse | CAG CTT GCT TGC TTA RAG CAA TAG GTC T ^1^ | 900 |
|  |  | BNP Probe | ABY - CCA GAY CTG GTC ATY GGA GCC CAA AAC TG - QSY ^1^ | 30 |
