# Supplementary material for: Diagnostic accuracy evaluation of a point-of-care antigen test for SARS-CoV-2 and influenza in UK primary care (RAPTOR-C19)
Source: PLoS One. 2025 Aug 7;20(8):e0329611. doi: 10.1371/journal.pone.0329611 (PMC12331028; doi:10.1371/journal.pone.0329611)
Supplement: S1 File — (DOCX) [file pone.0329611.s002.docx]

**S1 File. Subgroup analysis tables.**

**Table A.** Subgroup table for POCT SARS-CoV-2.

| **Subgroup** | **TP** | **FN** | **FP** | **TN** |
| --- | --- | --- | --- | --- |
| All participants | 19 | 8 | 3 | 451 |
| Male | 8 | 2 | 3 | 185 |
| Female | 11 | 6 | 0 | 266 |
| Adults (age 16+ years) | 16 | 5 | 3 | 282 |
| Age < 16 years | 3 | 3 | 0 | 169 |
| Age 16-39 years | 5 | 4 | 3 | 149 |
| Age 40-59 years | 8 | 0 | 0 | 93 |
| Age 60+ years | 3 | 1 | 0 | 40 |
| Ethnicity: White | 14 | 6 | 2 | 313 |
| Ethnicity: Asian | 3 | 1 | 1 | 82 |
| Ethnicity: Black | 1 | 1 | 0 | 20 |
| Ethnicity: All others | 1 | 0 | 0 | 36 |
| Main symptoms: none | 0 | 0 | 0 | 16 |
| Main symptoms: one | 3 | 0 | 0 | 140 |
| Main symptoms: two | 8 | 7 | 1 | 153 |
| Main symptoms: three | 1 | 1 | 2 | 73 |
| Main symptoms: four | 3 | 0 | 0 | 42 |
| Main symptoms: five | 4 | 0 | 0 | 27 |

TP: true positive, FN: false negative, FP: false positive, TN: true negative. Main symptoms were counted as the number of the following symptoms that participants reported with onset within 14 days before the recruitment date: fever, cough, fatigue, headache, sore throat.

**Table B.** Subgroup table for Influenza A.

| **Subgroup** | **TP** | **FN** | **FP** | **TN** |
| --- | --- | --- | --- | --- |
| All participants | 16 | 39 | 6 | 420 |
| Male | 9 | 15 | 2 | 172 |
| Female | 7 | 24 | 4 | 248 |
| Adults (age 16+ years) | 8 | 22 | 4 | 272 |
| Age < 16 years | 8 | 17 | 2 | 148 |
| Age 16-39 years | 5 | 16 | 1 | 139 |
| Age 40-59 years | 2 | 5 | 2 | 92 |
| Age 60+ years | 1 | 1 | 1 | 41 |
| Ethnicity: White | 9 | 27 | 4 | 295 |
| Ethnicity: Asian | 5 | 9 | 2 | 71 |
| Ethnicity: Black | 0 | 1 | 0 | 21 |
| Ethnicity: All others | 2 | 2 | 0 | 33 |
| Main symptoms: none | 0 | 0 | 0 | 16 |
| Main symptoms: one | 0 | 6 | 0 | 137 |
| Main symptoms: two | 7 | 20 | 4 | 138 |
| Main symptoms: three | 3 | 9 | 1 | 64 |
| Main symptoms: four | 3 | 1 | 1 | 40 |
| Main symptoms: five | 3 | 3 | 0 | 25 |

TP: true positive, FN: false negative, FP: false positive, TN: true negative. Main symptoms were counted as the number of the following symptoms that participants reported with onset within 14 days before the recruitment date: fever, cough, fatigue, headache, sore throat.

**Table C.** Subgroup table for Influenza B.

| **Subgroup** | **TP** | **FN** | **FP** | **TN** |
| --- | --- | --- | --- | --- |
| All participants | 2 | 7 | 9 | 463 |
| Male | 1 | 1 | 2 | 194 |
| Female | 1 | 6 | 7 | 269 |
| Adults (age 16+ years) | 2 | 4 | 5 | 295 |
| Age < 16 years | 0 | 3 | 4 | 168 |
| Age 16-39 years | 2 | 2 | 1 | 156 |
| Age 40-59 years | 0 | 2 | 2 | 97 |
| Age 60+ years | 0 | 0 | 2 | 42 |
| Ethnicity: White | 2 | 4 | 5 | 324 |
| Ethnicity: Asian | 0 | 1 | 2 | 84 |
| Ethnicity: Black | 0 | 0 | 2 | 20 |
| Ethnicity: All others | 0 | 2 | 0 | 35 |
| Main symptoms: none | 0 | 0 | 0 | 16 |
| Main symptoms: one | 0 | 0 | 2 | 141 |
| Main symptoms: two | 0 | 4 | 5 | 160 |
| Main symptoms: three | 1 | 1 | 0 | 75 |
| Main symptoms: four | 1 | 0 | 2 | 42 |
| Main symptoms: five | 0 | 2 | 0 | 29 |

TP: true positive, FN: false negative, FP: false positive, TN: true negative. Main symptoms were counted as the number of the following symptoms that participants reported with onset within 14 days before the recruitment date: fever, cough, fatigue, headache, sore throat.

**Table D.** Subgroup by CRN region for SARS-CoV-2.

| **Subgroup** | **TP** | **FN** | **FP** | **TN** |
| --- | --- | --- | --- | --- |
| All participants | 19 | 8 | 3 | 451 |
| Region 1 | 6 | 0 | 1 | 101 |
| Region 2 | 1 | 2 | 0 | 15 |
| Region 3 | 1 | 0 | 0 | 25 |
| Region 4 | 3 | 0 | 0 | 53 |
| Region 5 | 2 | 2 | 0 | 36 |
| Region 6 | 1 | 0 | 0 | 3 |
| Region 7 | 4 | 4 | 2 | 201 |
| Region 8 | 1 | 0 | 0 | 17 |

**Table E.** Subgroup by CRN region for Influenza A.

| **Subgroup** | **TP** | **FN** | **FP** | **TN** |
| --- | --- | --- | --- | --- |
| All participants | 16 | 39 | 6 | 420 |
| Region 1 | 5 | 7 | 1 | 95 |
| Region 2 | 0 | 0 | 0 | 18 |
| Region 3 | 2 | 1 | 1 | 22 |
| Region 4 | 1 | 4 | 1 | 50 |
| Region 5 | 2 | 1 | 2 | 35 |
| Region 6 | 0 | 0 | 0 | 4 |
| Region 7 | 5 | 25 | 1 | 180 |
| Region 8 | 1 | 1 | 0 | 16 |

**Table F.** Subgroup by CRN region for Influenza B.

| **Subgroup** | **TP** | **FN** | **FP** | **TN** |
| --- | --- | --- | --- | --- |
| All participants | 2 | 7 | 9 | 463 |
| Region 1 | 1 | 3 | 4 | 100 |
| Region 2 | 0 | 0 | 0 | 18 |
| Region 3 | 0 | 0 | 0 | 26 |
| Region 4 | 0 | 1 | 1 | 54 |
| Region 5 | 1 | 0 | 0 | 39 |
| Region 6 | 0 | 0 | 0 | 4 |
| Region 7 | 0 | 2 | 4 | 205 |
| Region 8 | 0 | 1 | 0 | 17 |
